# Differential Diagnosis Between Psychogenic Pseudosyncope and Vasovagal Syncope in Children: A Quantitative Scoring Model Based on Clinical Manifestations

**DOI:** 10.3389/fcvm.2022.839183

**Published:** 2022-01-27

**Authors:** Changjian Li, Yong Zhang, Ying Liao, Lu Han, Qingyou Zhang, Jia Fu, Dan Zhou, Shuai Long, Hong Tian, Hongfang Jin, Junbao Du

**Affiliations:** ^1^Department of Pediatrics, Peking University First Hospital, Beijing, China; ^2^Department of Cardiology, Wuhan Children's Hospital (Wuhan Maternal and Child Healthcare Hospital), Tongji Medical College, Huazhong University of Science and Technology, Wuhan, China; ^3^Cardiovascular Center, Children's Hospital, Fudan University, Shanghai, China; ^4^Key Laboratory of Molecular Cardiovascular Sciences, The Ministry of China, Beijing, China

**Keywords:** psychogenic pseudosyncope, vasovagal syncope, differential diagnosis, scoring model, binary logistic regression

## Abstract

The study was designed to explore a clinical manifestation-based quantitative scoring model to assist the differentiation between psychogenic pseudosyncope (PPS) and vasovagal syncope (VVS) in children. In this retrospective case-control study, the training set included 233 pediatric patients aged 5–17 years (183 children with VVS and 50 with PPS) and the validation set consisted of another 138 patients aged 5–15 years (100 children with VVS and 38 with PPS). In the training set study, the demographic characteristics and clinical presentation of patients were compared between PPS and VVS. The independent variables were analyzed by binary logistic regression, and the score for each variable was given according to the approximate values of odds ratio (OR) to develop a scoring model for distinguishing PPS and VVS. The cut-off scores and area under the curve (AUC) for differentiating PPS and VVS cases were calculated using receiver operating characteristic (ROC) curve. Then, the ability of the scoring model to differentiate PPS from VVS was validated by the true clinical diagnosis of PPS and VVS in the validation set. In the training set, there were 7 variables with significant differences between the PPS and VVS groups, including duration of loss of consciousness (DLOC) (*p* < 0.01), daily frequency of attacks (*p* < 0.01), BMI (*p* < 0.01), 24-h average HR (*p* < 0.01), upright posture (*p* < 0.01), family history of syncope (*p* < 0.05) and precursors (*p* < 0.01). The binary regression analysis showed that upright posture, DLOC, daily frequency of attacks, and BMI were independent variables to distinguish between PPS and VVS. Based on the OR values of each independent variable, a score of 5 as the cut-off point for differentiating PPS from VVS yielded the sensitivity and specificity of 92.0% and 90.7%, respectively, and the AUC value was 0.965 (95% confidence interval: 0.945–0.986, *p* < 0.01). The sensitivity, specificity, and accuracy of this scoring model in the external validation set to distinguish PPS from VVS were 73.7%, 93.0%, and 87.7%, respectively. Therefore, the clinical manifestation-based scoring model is a simple and efficient measure to distinguish between PPS and VVS.

## Introduction

Syncope is the inability to maintain an autonomous body position due to recoverable whole-brain hypoperfusion and manifests as a transient loss of consciousness (TLOC) (1). It is typically characterized by spontaneous and complete recovery of TLOC within a short period of time (2, 3). Vasovagal syncope (VVS) accounts for about 60–70% of syncope in children and adolescents (4). Psychogenic pseudosyncope (PPS) is the other entity of TLOC without virtual cerebral hypoperfusion or impaired physiological function (5–7). It is considered a conversion disorder in nature (8). The prevalence of PPS varies from 0 to 12%, with an average incidence rate of 4% (9–11). PPS and VVS share several similarities in clinical manifestations, such as recurrent episodes of TLOC and falls usually without convulsions. Since children with PPS have no convulsion-like symptoms, their attacks are sometimes considered as syncope in the preliminary evaluation in many cases. In a study including both adults and children as study subjects, even up to 50% of PPS cases were misdiagnosed as VVS at the beginning (9). In addition, the manifestation of some children with PPS may be taken as malingering attacks and the actual diagnosis is ignored due to the uneven understanding of PPS among medical institutions at all levels (12). Therefore, increasing reports suggested that the incidence of PPS in children may be underestimated (13–15). Furthermore, although PPS and VVS are similar in clinical manifestations, the management strategies and prognosis of PPS are completely different from those of VVS. The above facts suggest the absolute necessity of distinguishing PPS from VVS.

At present, there has not been any acknowledged clinical manifestation-based systematic procedure to differentiate PPS and VVS in children. Head-up tilt test (HUTT) has been performed to clarify the cause of syncope and is one of the auxiliary examinations to distinguish between VVS and PPS (16, 17). However, under many circumstances, the response of patients to HUTT alone is not sufficient enough to confirm the diagnosis of VVS as its sensitivity in the diagnosis is low (18). Therefore, several guidelines emphasized that it is important to explain the results of HUTT together with the clinical manifestations and make careful differentiation (19, 20). In addition, there are some limitations of the HUTT use. For example, the basic HUTT sometimes takes 45 min (min), and the drug-provocated HUTT is extended by another 20 min under certain circumstances (19). Furthermore, during HUTT, patients may sometimes present as cardiac arrest (21). Even after careful evaluation, the HUTT for pediatric patients may be suspended just because the child cannot cooperate very well and it is not being widely used in grassroots hospitals or even in some general hospitals (17, 22). Therefore, a simple, efficient, and rapid measure for the differentiation between PPS and VVS based on clinical manifestations is urgently needed.

Previous studies have shown that the episodes of unconsciousness in children with PPS usually last for a longer time (from 5 to 20 min or longer) and occur more frequently than those in children with VVS (11). Other clinical characteristics indicating the diagnosis of PPS include closing eyes without being pale look, no sweating during the attack, and seldom physical injury (20, 23). However, how to quantify these various clinical features and use them to discriminate between PPS and VVS is an urgent issue in clinical practice. In a previous study, the authors described a model composing of the posture during an episode, loss of consciousness (LOC) duration, and electrocardiogram-derived QT dispersion (24). However, the result of QT dispersion cannot be determined in a very quick and convenient way in grassroots hospitals.

Therefore, the present study was undertaken to develop a new scoring model to differentiate between PPS and VVS using recognized clinical features to help pediatricians, especially those working in grassroots hospitals, to differentiate pediatric PPS from VVS in a simple and rapid way.

## Methods

### Subjects

Totally, 233 children hospitalized in the Department of Pediatrics, Peking University First Hospital, China, from January 2012 to June 2021 were included in the training set. Of whom, 183 children (71 males and 112 females) had VVS with a median age of 11.0 (9.0, 13.0) years, and 50 children (22 males and 28 females) had PPS with a median age of 12.0 (9.0, 13.0) years. An additional 138 children treated at the Cardiovascular Center, Children's Hospital, Fudan University, China, from January 2009 to June 2021 were included in the external validation set, of whom 100 children (47 males and 53 females) had VVS with a median age of 10.0 (8.0, 12.0) years and 38 children (24 males and 14 females) had PPS with a median age of 11.0 (9.0, 13.0) years.

The diagnostic criteria of VVS are: (1) occurring primarily in older children and adolescents; (2) often accompanied by precipitating factors such as long periods of uprightness, mental tension, and sultry environment; (3) a clear history or aura of syncope; (4) a positive HUTT test; and (5) exclusion of other diseases such as cardiogenic, cerebrovascular or metabolic diseases (19, 20). The diagnostic criteria for PPS are based on the Diagnostic and Statistical Manual of Mental Disorders, Fifth Edition (DSM-V) (25).

The inclusion criteria of the study subjects: (1) those diagnosed as VVS or PPS; (2) patients under the age of 18 years old; (3) patients with normal routine biochemistry and 24-h Holter recordings results; (4) the data of the first confirmed hospitalization were included in the study for those with multiple hospitalizations; and (5) the children did not receive medication within 2 weeks.

Exclusion criteria of research subjects: (1) syncope caused by cardiogenic, cerebrovascular, and other diseases; (2) patients with non-sinus rhythm in electrocardiogram (ECG); (3) patients with incomplete medical records, and loss of data; (4) the children without HUTT examination; (5) patients diagnosed as PPS with VVS.

This study was approved by the Ethics Committee of Peking University First Hospital (2021–424) and Children's Hospital of Fudan University (2021–476), and the informed consent was permitted to be waived.

### Data Collection

We collected the demographic data of all the participants based on the medical records during their hospitalizations, including sex, age, and body mass index (BMI). The clinical manifestations as triggers or predisposing factors (e.g., upright posture, emotional stress, and stuffy environment), precursors, duration of loss of consciousness (DLOC), the daily frequency of attacks (the highest number of LOC episodes within 1 day, at least once) in the present history and the family history of syncope were collected. The upright posture means that the attacks happen when the patient is at an orthostatic posture, including standing for a long time and/or just standing up suddenly. Other kinds of situations were non-upright posture, for example, postures except orthostatism or walking, and/or exercising in the upright position. DLOC referred to the maximum recorded duration of the real or apparent LOC reported by the witness according to the medical records. We defined a child with a history of syncope in the family within two generations as having a positive family history. Besides, the baseline data of resting HR, resting systolic blood pressure (SBP), and resting diastolic blood pressure (DBP) as well as the 24-h average HR in Holter monitoring records of the patients were also recorded. The above data were obtained from the Medical Recording Management Digital System (Kaihua, Beijing, China). The medical history and laboratory findings of all the patients were reviewed in detail and recorded by a specialized investigator. The records were carefully proofread by another investigator independently.

### Methodology of HUTT and Dynamic Electrocardiogram

Children fasted for at least 4 h before the testing, stopped any vasoactive medication for at least five half-lives, and avoided the drink that could affect autonomic nervous system function (e.g., coffee). The test was performed in the morning, and the environment was kept quiet and dimly-lit at a suitable room temperature. Children first laid on the tilt table (SHUT-100A, Standard, Jiangsu and ST-711, Juchi, Beijing, China) for 10–30 min. During HUTT, HR, BP and ECG were recorded continuously with an ECG monitor (General Electric, New York, USA) and Finapres Medical System (FinometerPRO, FMS, The Netherlands). After the stabilization of HR and BP, the table was tilted upward at 60° and HR, BP, and ECG were continuously monitored till the positive response appeared, or otherwise till the whole test duration (45 min) if no positive response was observed. Positive response criteria of HUTT are listed below: (1) significant blood pressure drop (i.e., SBP ≤ 80 mmHg, DBP ≤ 50 mmHg, or ≥25% decrease in mean BP); (2) bradycardia (i.e., HR <75 bpm for children at 4–6 years of age, <65 bpm for children at 6–8 years of age, and <60 bpm for children at 8 years of age and older); (3) the presence of sinus arrest, premature junctional contractions; or (4) transient second-degree or higher atrioventricular block or cardiac arrest ≥3 s (19, 26, 27).

A 24-h ECG was recorded with an ECG recorder (Mortara Instrument, Milwaukee, Wisconsin, USA) and coffee, tea, or other drugs and strenuous exercise were avoided during the 24-h ECG. The 24-h ECG results were automatically analyzed by Mortara software (Mortara H-Scribe 7.0, Milwaukee, Wisconsin, USA) to obtain the 24-h average HR after automatic analysis.

### Statistical Analysis

All statistical analyses were performed using SPSS version 25.0 (IBM, New York, USA). The normality test of continuous variables was performed using the Shapiro–Wilk test. For data where both groups obeyed a normal distribution, the measured data were expressed as (*x* ± s) and the *t*-test was used to compare between the two groups. Non-normally distributed data were described as median (25th percentile, 75th percentile) and the differences between groups were compared using the Mann-Whitney *U* test. The categorical variables were described by frequency and constituent ratio, and comparisons between groups were made using chi-square tests.

To establish a scoring system for differential diagnosis, variables with a statistical difference of *p* < 0.05 in the univariate analysis of the comparison between the PPS and VVS groups were included in a binary logistic regression, and for clinical application, continuous variables were transformed into dichotomous variables using cut-off values extracted from receiver operating characteristic (ROC) curves. Each variable derived from the regression was given a score according to the approximate odds ratio (OR) values, and the total score of a patient was calculated by adding up the scores of all identified variables, forming a scoring model. The Hosmer-Lemeshow test was used to assess the goodness of fit of the discriminant model. The ROC curve was performed to assess the power of the above scoring model in the differential diagnosis and determine the optimal cut-off score based on the maximum Youden index. Finally, the sensitivity, specificity, and accuracy of the scoring model were evaluated in the differentiation between PPS and VVS in an external verification study. A *p*-value <0.05 was considered significant.

## Results

### Demographic Features

In the training set, 183 and 50 children were included in the VVS and PPS groups, respectively. The two groups did not show any statistically significant difference in sex, age, resting HR, resting SBP, and resting DBP (*p* > 0.05). Children in the PPS group had a much higher BMI (21.0 kg/m^2^ vs. 17.7 kg/m^2^) and 24-h average HR (84.0 bpm vs. 81.0 bpm) than those in VVS group, and the differences were statistically significant (*p* < 0.01, Table 1).

**Table 1:** Comparison of the demographic characteristics between the VVS and PPS groups in training set.

**Groups**	**VVS**	**PPS**	***t/Z/x* ^2^**	***p*-value**
Patients (n)	183	50		
Age (y)	11.0 (9.0, 13.0)	12.0 (9.0, 13.0)	−0.489	0.625
Sex (M/F)	71/112 (38.8%/61.2%)	22/28 (44.0%/56.0%)	0.443	0.506
BMI (kg/m^2^)	17.7 (16.1, 20.0)	21.0 (17.0, 24.0)	−4.102	<0.01
Resting HR (bpm)	76.0 (68.0, 85.0)	79.5 (73.0, 86.3)	−1.839	0.066
Resting SBP (mmHg)	106.0 (98.0, 112.0)	108.0 (100.0, 116.3)	−1.578	0.115
Resting DBP (mmHg)	62.8 ± 7.2	64.9 ± 9.0	−1.733	0.085
24-h average HR (bpm)	81.0 (75.0, 89.0)	84.0 (79.0, 93.0)	−2.900	<0.01

*VVS, vasovagal syncope; PPS, psychogenic pseudosyncope; M/F, Male/Female; BMI, body mass index; HR, heart rate; bpm, beat per minute; SBP, systolic blood pressure; DBP, diastolic blood pressure*.

### Comparisons of Clinical Features Between PPS and VVS Groups

There was no statistical difference in emotional stress (*p* = 0.097) and stuffy environment (*p* = 0.096) before syncopal episode between the two groups; while, the significant differences were found in other manifestations, including DLOC (*p* < 0.01), daily frequency of attacks (*p* < 0.01), upright posture (*p* < 0.01), precursors (*p* < 0.01), and family history of syncope (*p* < 0.05, Table 2).

**Table 2:** Clinical features of patients diagnosed with VVS and PPS groups in training set.

**Groups**	**VVS**	**PPS**	***Z/x* ^2^**	***p*-value**
Patients (n)	183	50		
DLOC (min)	2.0 (1.0, 4.0)	20.0 (5.8, 60.0)	−7.205	<0.01
Daily frequency of attacks (times)	1 (1, 1)	1 (1, 3)	−7.763	<0.01
Upright posture (Yes/No)	165/18 (90.2%/9.8%)	13/37 (26.0%/74.0%)	89.655	<0.01
Stuffy environment (Yes/No)	37/146 (20.2%/79.8%)	5/45 (10.0%/90.0%)	2.775	0.096
Emotional stress (Yes/No)	26/157 (14.2%/85.8%)	12/38 (24.0%/76.0%)	2.759	0.097
FH of syncope (Yes/No)	34/149 (18.6%/81.4%)	2/48 (4.0%/96.0%)	6.390	0.011
Precursors (Yes/No)	132/51 (72.1%/27.9%)	22/28 (44.0%/56.0%)	13.868	<0.01

*VVS, vasovagal syncope; PPS, psychogenic pseudosyncope; DLOC, duration of loss of consciousness; min, minute; FH, family history*.

### The Cut-Off Value for Binary Classification of the Continuous Variables

Among the 7 variables showing statistical differences (*p* < 0.05) in comparison between PPS and VVS groups, including upright posture, DLOC, daily frequency of attacks, BMI, precursors, family history of syncope and 24-h average HR, there were 4 continuous variables (DLOC, daily frequency of attack, BMI and 24-h average HR) which were converted into dichotomous variables, respectively, for the ease of clinical application. The cut-off value, *p*-value, sensitivity and specificity of these continuous variables were shown in Table 3.

**Table 3:** The cut-off value for diclassification the continuous variables in training set.

**Test result variable (s)**	**Cut-off value**	**AUC (95% CI)**	***p*-value**	**Sensitivity**	**Specificity**
DLOC	≥9 min	0.824 (0.749, 0.900)	<0.01	0.740	0.863
Daily frequency of attacks	≥1.5 times^a^	0.705 (0.611, 0.800)	<0.01	0.440	0.962
BMI	≥20.5 kg/m^2^	0.689 (0.601, 0.778)	<0.01	0.580	0.792
24-h average HR	≥93 bpm	0.634 (0.549, 0.719)	<0.01	0.300	0.913

*AUC, Area under curve; CI, Confidence Interval; DLOC, duration of loss of consciousness; min, minute; BMI, body mass index; DBP, diastolic blood pressure; HR, heart rate; bpm, beat per minute*.

a *The cutoff value of daily frequency of attacks was defined as ≥twice because the actual number of syncope is an integer in clinical practice*.

### Building a Scoring Model to Identify PPS and VVS

Totally seven dichotomous variables were selected as independent variables for further logistic regression analysis using the backward conditional method, including upright posture (Yes/No), DLOC (≥9 min/ <9 min), daily frequency of attacks (≥twice/ < twice), BMI (≥20.5 kg/m^2^/ <20.5 kg/m^2^), precursors (Yes/No), family history of syncope (Yes/No) and 24-h average HR (≥93.0 bpm/ <93.0 bpm). Finally, four variables (upright posture, DLOC, daily frequency of attacks, and BMI) were determined as the independent variables to distinguish PPS from VVS. The statistical data of Hosmer-Lemeshow in each step did not show any significance (*p* > 0.05), suggesting that the goodness of fit was satisfactory.

According to the OR value of each independent variable, the score was assigned for each variable as follows (Table 4). (1) Upright posture: if there was no static upright posture as a predisposing factor before the TLOC event, four points were assigned, and otherwise, 0 point was assigned; (2) DLOC: if DLOC was ≥9 min, four points were assigned, and otherwise, 0 point was assigned; (3) daily frequency of attack: if daily frequency of attacks was ≥twice, 8 points were assigned, and otherwise, 0 point was assigned; and (4) BMI: if BMI was ≥20.5 kg/m^2^, 1 point was assigned, and otherwise, 0 point was assigned. The total score for the four variables was calculated for each patient in the PPS and VVS groups, respectively.

**Table 4:** Coefficients of binary logistic regression in training set.

**Variable (s)**	**Cut-off value**	***p*-value**	**Odds ratio (95% CI)**	**Points**
Upright posture	Yes/No	<0.01	24.390 (7.179, 82.861)	4
DLOC	9 min	<0.01	22.694 (6.257, 82.317)	4
Daily frequency of attacks	Twice	<0.01	49.476 (10.286, 238.407)	8
BMI	20.5 kg/m^2^	<0.01	5.974 (1.898, 18.801)	1

*CI, Confidence Interval; DLOC, duration of loss of consciousness; min, minute; BMI, body mass index*.

**Figure 1:**
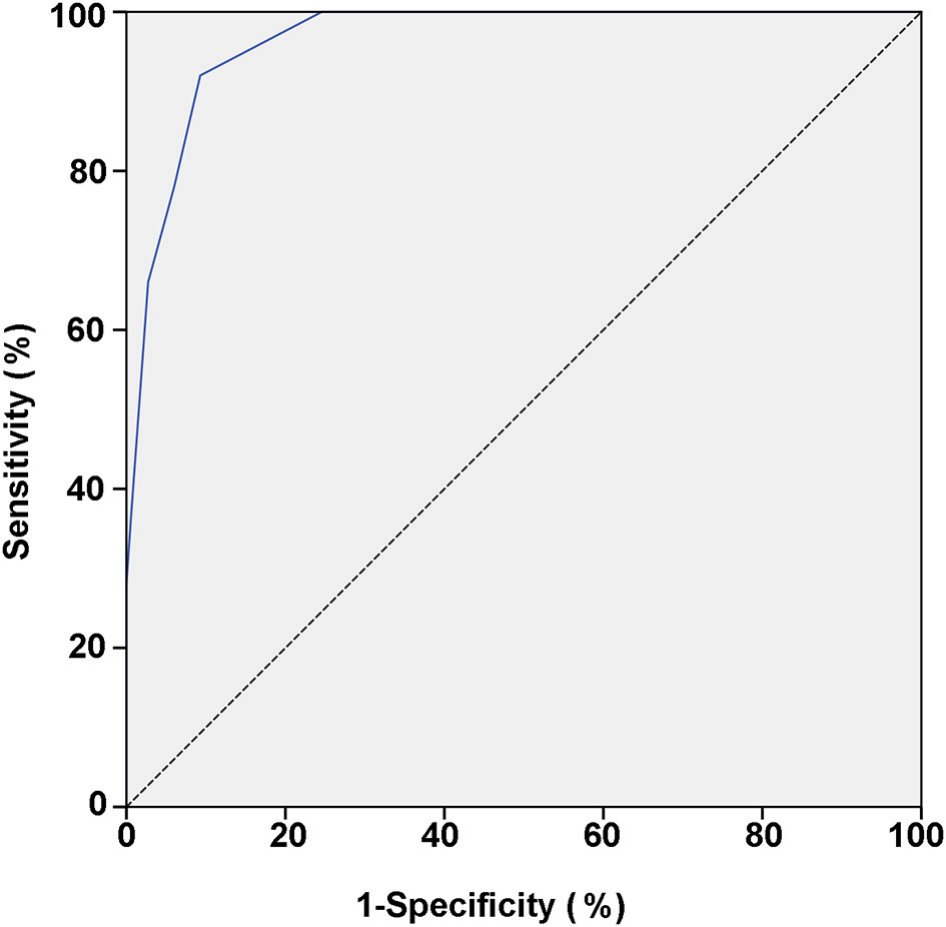
ROC curve of the scoring model between VVS and PPS groups. The vertical and horizontal axes of the curve represent predictive sensitivity and positivity (1-specificity), respectively. The 45° slash indicates that the sensitivity is equal to the false positive rate, indicating no predictive value. The blue curve represents the ROC curve of the scoring model for the predictive value of the PPS. The AUC represents the predicted value for different cut-off values and it has a value of 0.965 (95% CI: 0.945–0.986; *p* < 0.01). ROC, receiver operating characteristic; PPS, psychogenic pseudosyncope; VVS, vasovagal syncope; AUC, area under the curve; CI, confidence interval.

The power for differential diagnosis of the total score based on this model was assessed by the ROC curve. As a result, the area under the curve of the ROC was 0.965 (95% confidence interval: 0.945–0.986, *p* < 0.01). When the total score ≥5 points was used as the cut-off value for initial differentiation between pediatric PPS and VVS, its sensitivity was 92.0% and specificity was 90.7% for the diagnosis of PPS (Table 4; Figure 1).

### External Validation

To verify the efficiency of the scoring model, total scores of the patients with a definite clinical diagnosis of PPS or VVS in the validation set were calculated. Patients with total scores ≥5 points or <5 points were suspected to be PPS or VVS, respectively, according to the scoring model (Table 5). The suspected diagnosis based on the scoring model was then compared with the patient's true definitive clinical diagnosis, and the sensitivity, specificity, and accuracy of the scoring model in distinguishing between PPS and VVS were calculated to be 73.7%, 93.0%, and 87.7%, respectively.

**Table 5:** Predictive values of scoring model in external validation set.

**Score (point)**	**Clinical diagnosis**		**Total**
	**PPS**	**VVS**	
≥5	28	7	35
<5	10	93	103
Total	38	100	138

*VVS, vasovagal syncope; PPS, psychogenic pseudosyncope*.

## Discussion

PPS and VVS share many similar clinical characteristics, but they are completely different diseases. This study showed that there were significant differences between the two diseases in DLOC, daily frequency of attacks, upright posture, family history of syncope, precursors, BMI, and 24-h average HR. The scoring model for the preliminary discrimination between PPS and VVS by four variables (daily frequency of attacks, upright posture, DLOC, and BMI) was established through binary logistic regression. By assigning scores for the four variables according to the OR values, we determined the final model with a maximum total score of 17 points. When the total score of the child was ≥5 points, the sensitivity and specificity for the possible differentiation of PPS from VVS were 92.0% and 90.7%, respectively.

In this study, the daily frequency of attacks ≥twice had the highest weight (eight points) in indicating the diagnosis of PPS, followed by DLOC ≥9 min (four points) and onset without predisposing upright posture (four points). In other words, children with PPS were more likely to experience more than one event within 1 day and have a longer duration of each episode. The median of DLOC in the PPS group reached 20 min in this study. The difference in frequency and DLOC between PPS and VVS are closely related to their distinct pathogenesis. A PPS-like event can occur anytime and anywhere with or without psychological triggers, which may vary from person to person and the duration may also differ in length without the challenge of cerebral ischemia. As for VVS, the syncopal events often occur under specific inducement and the events are usually not that frequent (28). Although VVS is sometimes characterized by cluster attacks, the recurrences usually occur a few days later after the first episode (29). Regarding the posture as a predisposing factor of attacks, children in the VVS group were more likely to have an event while standing upright than those in the PPS group (90.2 vs. 26.0%, *p* < 0.01). VVS is an important form of acute orthostatic intolerance. When standing, the peripheral blood volume in the abdomen and lower limbs increases due to the gravity; therefore, when paradoxical vagal activation occurs in children with VVS during the adaptation to upright posture, a decrease in blood return volume causes a sharp drop in cardiac output, leading to syncope (28). In contrast, the mechanism for PPS attack is not directly related to the posture, and children with PPS may faint at any posture. Finally, BMI ≥20.5 kg/m^2^ was assigned as 1 point to cue the diagnosis of PPS. Some scholars found that children with VVS had a lower BMI than healthy controls (30). This may be due to the fact that children with low BMI have relatively lower blood volume, which may deteriorate orthostatic intolerance (31). However, the relationship between BMI and PPS is not clear. Collectively, the four variables derived from the binary logistic regression equation can reflect the distinct characteristics between pediatric PPS and VVS.

In the external validation, the sensitivity of the scoring model is not as high as that of the training set in our study. The reason may be the fact that BMI and clinical characteristics of children in different regions may not be that identical. In addition, the DLOC is difficult to be reported exactly because the duration is often estimated by the witness. Therefore, the large sample sizes and multicenter studies are still necessary to improve the efficiency of the discriminant model.

In the previous clinical differential diagnosis between PPS and VVS, no clinical manifestations are specific and the weight of each feature is not clear. Therefore, pediatricians can only analyze the clinical data and make judgment according to their own experiences. HUTT is an important examination to identify the causes of syncope, but as mentioned above, its value in distinguishing between PPS and VVS is limited (32).

In this present study, several features with the most distinguishing significance were analyzed, and these variables were quantified according to the weight assignment. By this scoring model, a rapid preliminary judgment between PPS and VVS can be made just through a simple inquiry of medical history and basic measurement of height and weight, which is highly practical and easy to be popularized. Of course, in the diagnostic procedure of TLOC, it is also necessary to identify other causes except for PPS and VVS. Nevertheless, the significance of this study is that, contrary to the relatively complex diagnostic procedure at present, doctors at different levels can obtain a preliminary judgment and make a more targeted investigation plan for children suffering from TLOC with the help of our scoring model. If the total score indicates PPS, the patient should be recommended to see the psychiatric specialists for further evaluation while screening for other physical diseases, to manage the patient in a more efficient and comprehensive way. While, if the total score suggests VVS, the patient should be recommended to have HUTT, etc. Therefore, the results of this study will be helpful to suggest reasonable referrals and optimize the differential diagnostic process in clinical practice. Furthermore, compared with the previous study (24), this scoring model, for the first time, used the clinical manifestations only instead of doing a variety of laboratory investigations at the initial diagnostic step, and the newly developed scoring model yielded relatively high sensitivity and specificity for differentiating PPS from VVS.

Our study also had some limitations. Children with VVS were unable to accurately describe the frequency and duration of syncope, and the information of syncopal attack was sometimes described by their family members or bystanders. In the training set, we included children aged 5–17 years, and in the validation set, we recruited the subjects aged 5–15 years, although the diagnostic criteria were kept the same. The study was a retrospective study, in which only hospitalized children were included. Therefore, prospective, multicenter and large sample-sized studies are still necessary to optimize this model. Nevertheless, in the present study, we developed a useful, easy-to-operate, and very inexpensive clinical characteristics-based scoring model for pediatricians to make a quick and initial differentiation between PPS and VVS in children.

## Conclusion

This study developed a clinical manifestation-based scoring model to differentiate PPS from VVS, assisting in making a quick initial differential diagnosis. Further multicenter studies are still needed to improve the ability to differentially diagnose pediatric TLOC cases.

## Data Availability Statement

The raw data supporting the conclusions of this article will be made available by the authors, without undue reservation.

## Ethics Statement

The studies involving human participants were reviewed and approved by the Ethics Committee of Peking University First Hospital and Children's Hospital of Fudan University. Written informed consent for participation was not provided by the participants' legal guardians/next of kin because the required informed consent was permitted to be waived by the Ethics Committee of Peking University First Hospital and Children's Hospital of Fudan University.

## Author Contributions

YZ, HJ, JD, and HT presented thesis ideas and contributed to discussion. CL, YL, JD, HJ, and QZ assisted in collecting and analyzing data. HT, LH, and SL provided external validation data and performed analysis. JF and DZ contributed to discussion. CL, YL, and LH wrote the original manuscript. JD, YZ, HJ, and HT critically reviewed and proofread the manuscript. All authors have read and agreed to submit the manuscript.

## Funding

This study was supported by Peking University Clinical Scientist Program (BJMU2019LCKXJ001) and the Fundamental Research Funds for the Central Universities.

## Conflict of Interest

The authors declare that the research was conducted in the absence of any commercial or financial relationships that could be construed as a potential conflict of interest.

## Publisher's Note

All claims expressed in this article are solely those of the authors and do not necessarily represent those of their affiliated organizations, or those of the publisher, the editors and the reviewers. Any product that may be evaluated in this article, or claim that may be made by its manufacturer, is not guaranteed or endorsed by the publisher.
